# The Complexity of Background Clutter Affects Nectar Bat Use of Flower Odor and Shape Cues

**DOI:** 10.1371/journal.pone.0136657

**Published:** 2015-10-07

**Authors:** Nathan Muchhala, Diana Serrano

**Affiliations:** 1 Department of Biology, University of Missouri St. Louis, St. Louis, Missouri, United States of America; 2 Escuela de Ciencias Biologicas, Pontificia Universidad Católica del Ecuador, Quito, Ecuador; University of Southern Denmark, DENMARK

## Abstract

Given their small size and high metabolism, nectar bats need to be able to quickly locate flowers during foraging bouts. Chiropterophilous plants depend on these bats for their reproduction, thus they also benefit if their flowers can be easily located, and we would expect that floral traits such as odor and shape have evolved to maximize detection by bats. However, relatively little is known about the importance of different floral cues during foraging bouts. In the present study, we undertook a set of flight cage experiments with two species of nectar bats (*Anoura caudifer* and *A*. *geoffroyi*) and artificial flowers to compare the importance of shape and scent cues in locating flowers. In a training phase, a bat was presented an artificial flower with a given shape and scent, whose position was constantly shifted to prevent reliance on spatial memory. In the experimental phase, two flowers were presented, one with the training-flower scent and one with the training-flower shape. For each experimental repetition, we recorded which flower was located first, and then shifted flower positions. Additionally, experiments were repeated in a simple environment, without background clutter, or a complex environment, with a background of leaves and branches. Results demonstrate that bats visit either flower indiscriminately with simple backgrounds, with no significant difference in terms of whether they visit the training-flower odor or training-flower shape first. However, in a complex background olfaction was the most important cue; scented flowers were consistently located first. This suggests that for well-exposed flowers, without obstruction from clutter, vision and/or echolocation are sufficient in locating them. In more complex backgrounds, nectar bats depend more heavily on olfaction during foraging bouts.

## Introduction

Finding appropriate food sources in forests with high biodiversity poses an important challenge to foraging animals. This is especially difficult for small animals with high metabolic rates like hummingbirds and glossophagine nectar-feeding bats (hereafter ‘nectar bats’), which require constant energy intake to meet their daily energy expenditures [1]. Selection on these animals will favor the ability to quickly locate floral resources. For their part, these animal-pollinated flowers also have a vested interest in being easily located by their pollinators. While plants in antagonistic plant-animal interactions (e.g. herbivory, seed predation) evolve to avoid detection, in pollination and seed-dispersal mutualisms selection favors traits that maximize detection. We have learned much about foraging behaviour for other pollinators (e.g., [2–5]), yet relatively little is known about nectar bat foraging. Studies of pollen carried on fur or in feces reveal which flowers nectar bats visit (e.g., [6–9]), but information on how bats locate flowers is much more difficult to obtain given their nocturnal habits, small size, and extremely rapid flower-visits (approximately 0.5 s; [10]).

One aspect of nectar bat foraging that is well understood is the importance of spatial memory. As demonstrated by various authors [11–14], spatial memory is well developed in nectar bats, allowing them to quickly relocate reliable food sources. Likely as an adaptation to such well-developed spatial memory, individual bat-adapted plants typically flower for months while presenting only one or few open flowers each night (‘steady-state flowering’ sensu [15]).

Spatial memory will aid in revisiting known flowers, but how do bats initially locate new floral resources? Echolocation, olfaction, and vision could all play important roles. Foraging microchiropteran bats typically rely heavily on echolocation, and nectar bats may be able to discriminate potential floral resources based on echoreflectance patterns [16, 17]. In fact, specialized dish-shaped leaves [18] and petals [19] have been shown to function as acoustic guides for bats, and the waxy petals common to flowers adapted to bat pollination (hereafter ‘bat flowers’) may similarly serve to amplify echo reflectance. The fact that bat flowers typically have long pedicels that present them well beyond the plant’s foliage may also represent an adaptation to improve detection via echolocation; in this case, by reducing background clutter around the flowers [20]. The potential importance of olfaction is implicated by the strong, musky scent typical of bat flowers [21, 22], and experiments show that glossophagine nectar bats have an innate preference for sulphuric scents [23]. Glossophagine olfactory bulbs and associated brain regions are relatively large [24, 25], further implicating the importance of olfaction for these animals. Vision is likely the least important of nectar bat senses for locating flowers, given that they forage at night. Nectar bats are color-blind, and bat flowers are typically dull-colored. However, nectar bats can discriminate between visual patterns at least as effectively as lab rats [26], and rely more on visual than acoustic cues during escape responses [27]. Additionally, they can perceive ultraviolet light, which may aid in navigating at dusk or dawn or in detecting ultraviolet reflected by flowers [28].

In evaluating the use of different sensory modalities, it is important to consider the background that the target is found in. For example, flowers may become more difficult to see in a particularly cluttered background of dense foliage. And for an echolocating animal, a dense background can create unwanted ‘clutter echoes’ (sensu [29]) which obscure the target. For insectivorous bats, background clutter around prey items has been found to lead to decreased reliance on echolocation and increased reliance on vision [30] and/or on prey-generated sounds [31, 32].

In this study, we explore the importance of different senses during nectar bat foraging. Specifically, we examined whether flower odor or shape are more important for locating new floral resources. To do this, we presented nectar bats in flight cages with artificial flowers of different odors and shapes, and eliminated the use of spatial memory during foraging by randomly shifting the flower positions after each flower visit. We also tested the importance of background clutter on foraging behaviour by performing experiments either with a simple background or with an array of foliage behind the flowers.

## Materials and Methods

**Fig 1:**
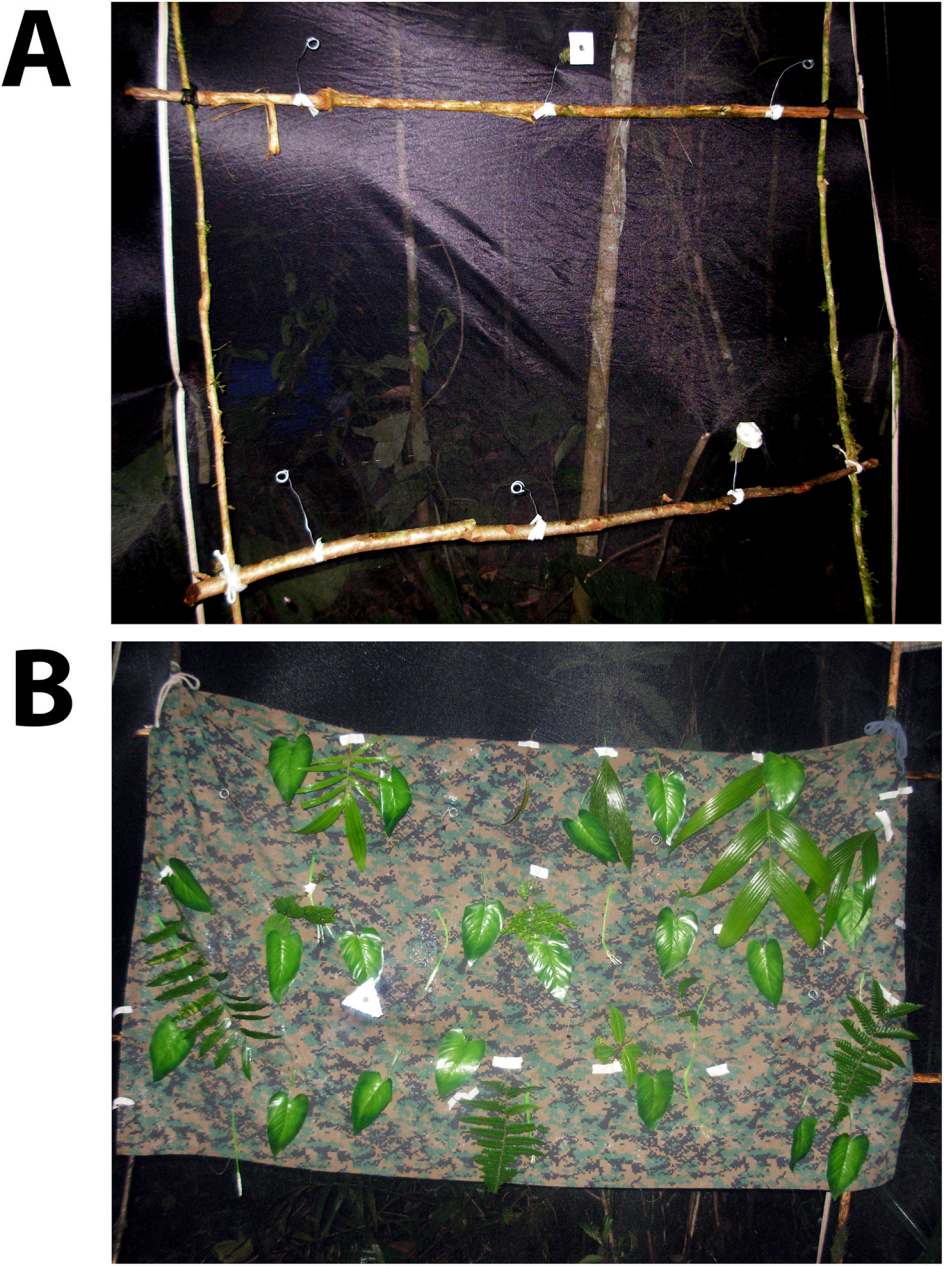
Experimental Set-up. A) Trellis used to position the artificial flowers, with a simple background and two artificial flowers, and B) trellis with a complex background.

This study was conducted from July 2010 to January 2011 during two-week visits to five cloud forest sites in Ecuador (S1 Table). We captured bats in mist nets set near chiropterophilous flowers and along paths in the forest. All bats were released immediately with the exception of six individuals of *Anoura caudifer* and six of *A*. *geoffroyi* (Chiroptera, Phyllostomidae, Glossophaginae). These were housed individually in screen tents (3 m^2^ x 2 m high) for two nights and maintained on a diet of 20% sugar solution *ad libitum* between experiments (and subsequently released after experiments). For the experimental setup, an artificial feeder array consisting of a trellis made from two vertical and two horizontal 1.6-m-long poles (Fig 1A) was placed inside the tent. Six wire holders for artificial flowers were placed along the horizontal poles, with 40 cm between them. We chose this distance of separation because bats have been shown to treat two feeders as different entities when they are greater than 30 cm apart, while they treat them as a single entity when closer than 30 cm (i.e. as different flowers within a single inflorescence; [12]).

Sugar-water was presented in artificial flowers which consisted of a test tube with an artificial corolla surrounding the opening. To ensure that experimental results were generalizable, we ran experiments with several different pairs of shapes and textures of corollas: 1) plastic circles vs. cloth pentagons, 2) styrofoam triangles vs. cardboard stars, and 3) foam squares vs. fabric tubes (S1 Fig). These corollas all had a similar surface area of approximately 9 cm^3^. We covered the test tube behind the corolla with a sleeve of coarse green cloth to minimize any echoacoustic or visual signals from the tube itself. Directly behind the corolla we also positioned a small compartment with or without a ball of cotton containing a scent solution. Two types of scents were used: either an artificial banana scent (Colorisa S.A.) or dimethyl disulfide (Merck Laboratory C.A.). The latter is a compound commonly found in bat-pollinated flowers [21, 23], and emits a sulphuric, skunk-like scent. Artificial flowers with scents were never re-used for scent-less flowers (that is, scent-less flowers had empty compartments which never previously had scent-filled cotton inside).

Each bat was exposed to a training phase followed by a series of experiments. During the training phase, the bat was presented with an artificial flower with a particular shape (of the six options) and scent (of the two options). The flower was located in one of the six possible array positions. After each bat visit, the flower was refilled with a 20% sucrose solution and randomly shifted to a different location in the array. The goal of this training phase was to accustom the bat to the particular shape/scent combination and to locating the flower without relying on spatial memory. To achieve the latter, we continued the training phase until the bat made five visits directly to a flower without first visiting the position in the array where the flower had previously been located. The time this training took varied between individuals from 0.6 to 3.33 hrs.

For the experiment, we presented bats with two flowers: one with the same shape as the training flower but without the odor, and the other with the same odor as the training flower but with a different shape. Thus this approach directly addresses the question: was the bat previously relying on odor or shape to locate the training flower as we shifted it around the array? These two flowers were placed at random positions on the array. The bat was allowed to make a single visit, after which we recorded which flower was visited, and then randomly shifted the location of both of the flowers again. We repeated this procedure until the bat made a total of 40 visits. Because preliminary results demonstrated that bats lose interest or appetite after too many visits, we divided this experiment into two phases: after 20 visits, bats were given a 10-minute ‘break’ without access to flowers, and then performed the final 20 visits. During both the training phase and the experiments, we used an Energizer 4-LED headlamp that could switch between dim white and dim red light; white light was used to shift locations of flowers, and red light was used to observe subsequent bat visitation.

We performed the experiments described above for two types of backgrounds, one simple and the other complex. We held each bat for two nights, using one night for complex-background experiments and the other for simple-background experiments. Each night consisted of a training phase and an experimentation phase, as outlined above, with half of the bats receiving the ‘complex’ treatment first, the other half the ‘simple’ treatment first. For the simple background, nothing was placed between the trellis feeder-array and the dark screen cloth of the experimental tent (Fig 1A). For the complex background, we added a camouflage-colored fabric covered in an assortment of natural and artificial foliage (Fig 1B). In all backgrounds, artificial flowers were presented on wires that positioned them approximately 8 cm in front of the trellis/fabric.

The dimethyl disulfide odor was used in experiments for half of the bats (e.g. three individuals of *Anoura caudifer* and three of *A*. *geoffroyi*), while the banana scent was used for the other half. The shapes tested during the experiments were: pentagonal vs. circular, square vs. tubular and triangle vs. star. Each pair of shapes was tested with two individuals of *Anoura caudifer* and two of *A*. *geoffroyi*, with the identity of the training shape different for the two individual bats (e.g. pentagonal training flowers followed by pentagonal and circle experimental flowers for one bat, circle experimental flowers followed by pentagonal and circle experimental flowers for the other bat).

We used a general linear model (GLM) to statistically analyse effects of the experimental factors on foraging behaviour. The response variable was the number of visits to the flower with the training-flower odor (rather than the one with the training-flower shape) out of the 20 visits recorded for each trial. Lower values for this response variable (<10) indicate reliance on shape, while higher values (>10) indicate reliance on odor. The GLM included four factors: 1) bat species (*Anoura caudifer* or *A*. *geoffroyi*), 2) scent used (banana or dimethyl disulfide), 3) background (simple or complex), and 4) trial (first set of 20 or second set of 20). The first two of these were between-subjects factors, while the last two were repeated-measures factors. A Levene’s test showed the data met the assumption of equal variance between groups, and Shapiro-Wilk tests found no significant deviations from normality. Analyses were performed in SPSS version 22.0.

### Ethics Statement

This research was approved by the Ministry of the Environment of Ecuador (permits 001-2010-B-DPMS/MAE and 006-IC-FAU/FLO-DPZCH/MA; note that the Pontificia Universidad Católica del Ecuador does not require a separate IACUC approval).

## Results

**Fig 2:**
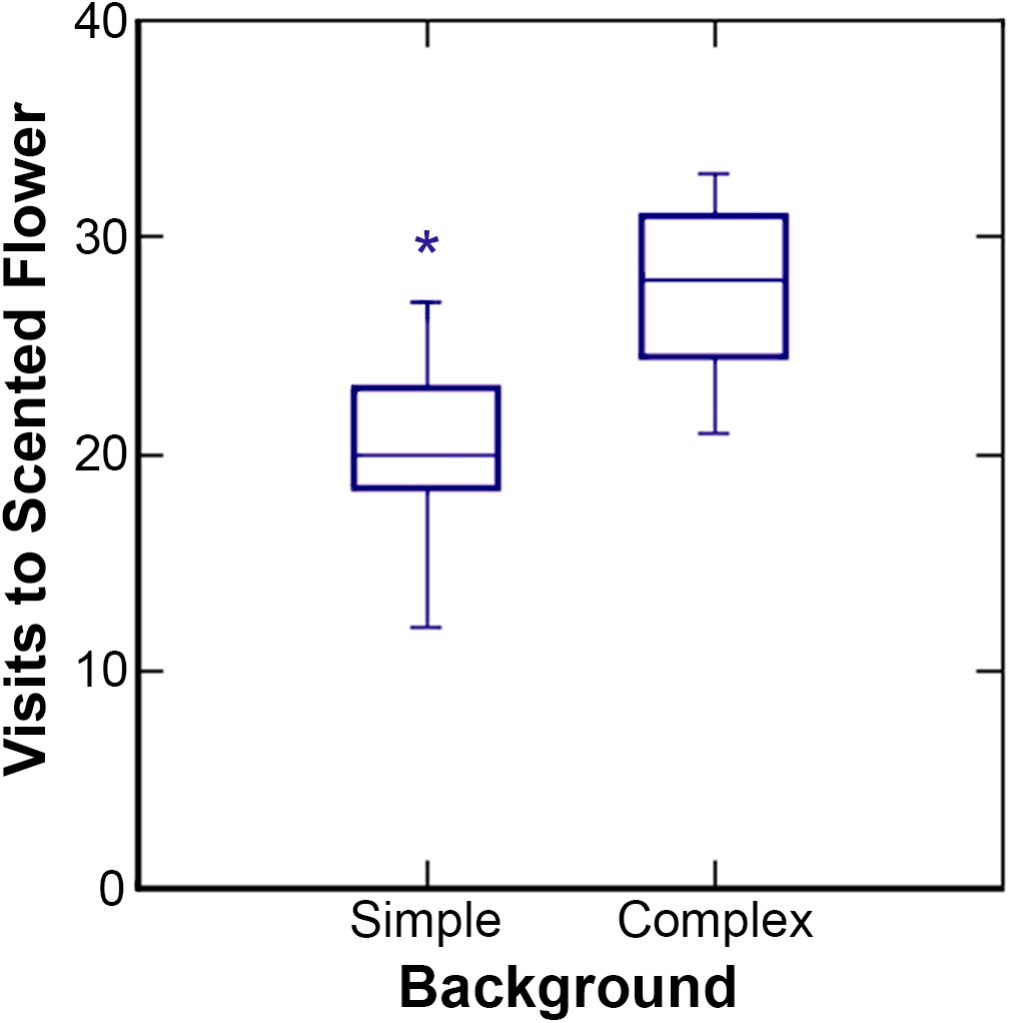
Main effect of background. Number of visits, of the 40 total, to the flower with the training-flower scent (versus the one the training-flower shape) for simple and complex backgrounds.

**Fig 3:**
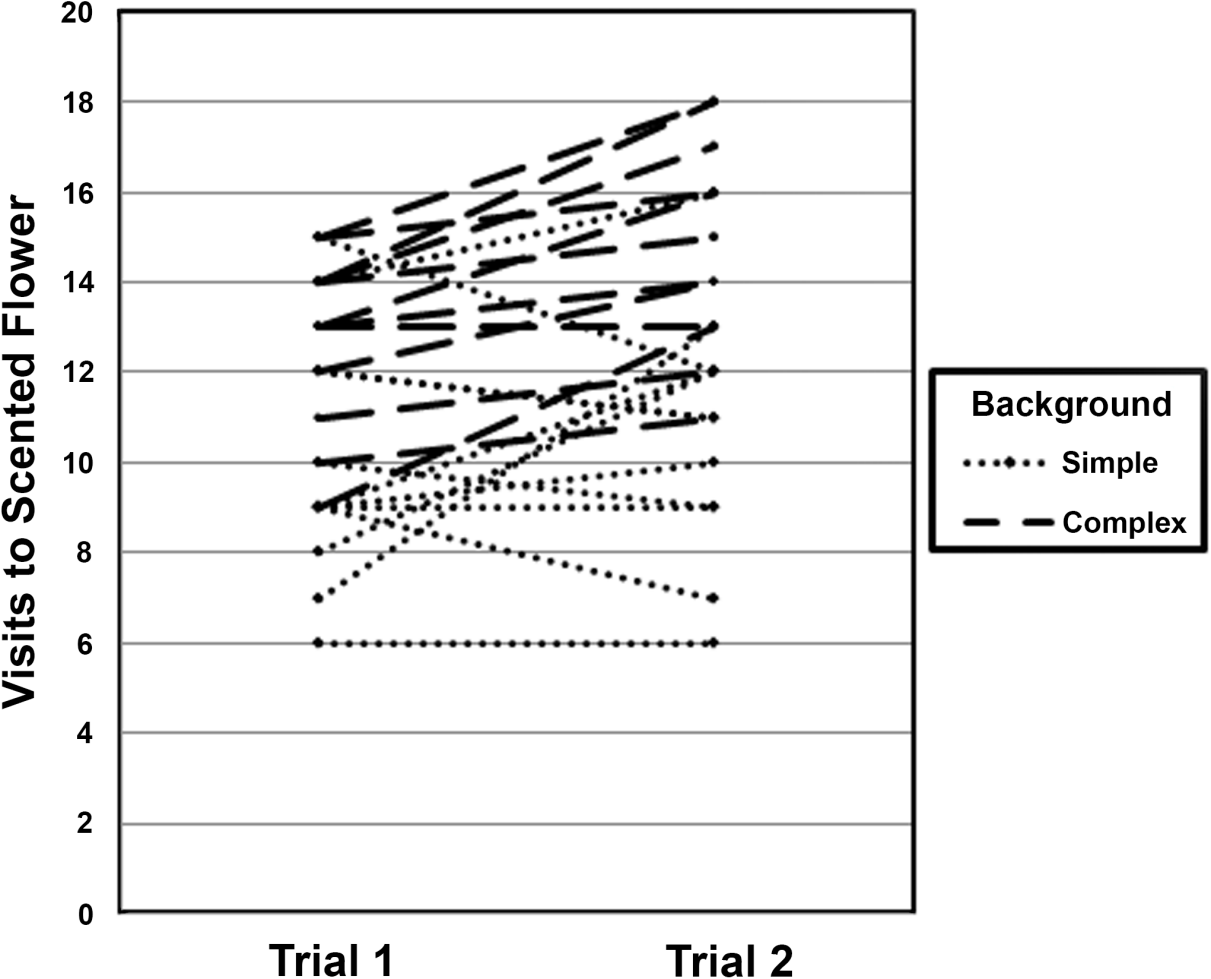
Main Effect of Trial Number. Number of visits to the flower with the training-flower scent (versus the one the training-flower shape) for the first set of 20 flower visits (trial 1) and the second set of 20 visits (trial 2). Lines represent individual bats, with dotted lines corresponding to experiments in simple backgrounds and dashed lines to experiments in complex backgrounds.

**Fig 4:**
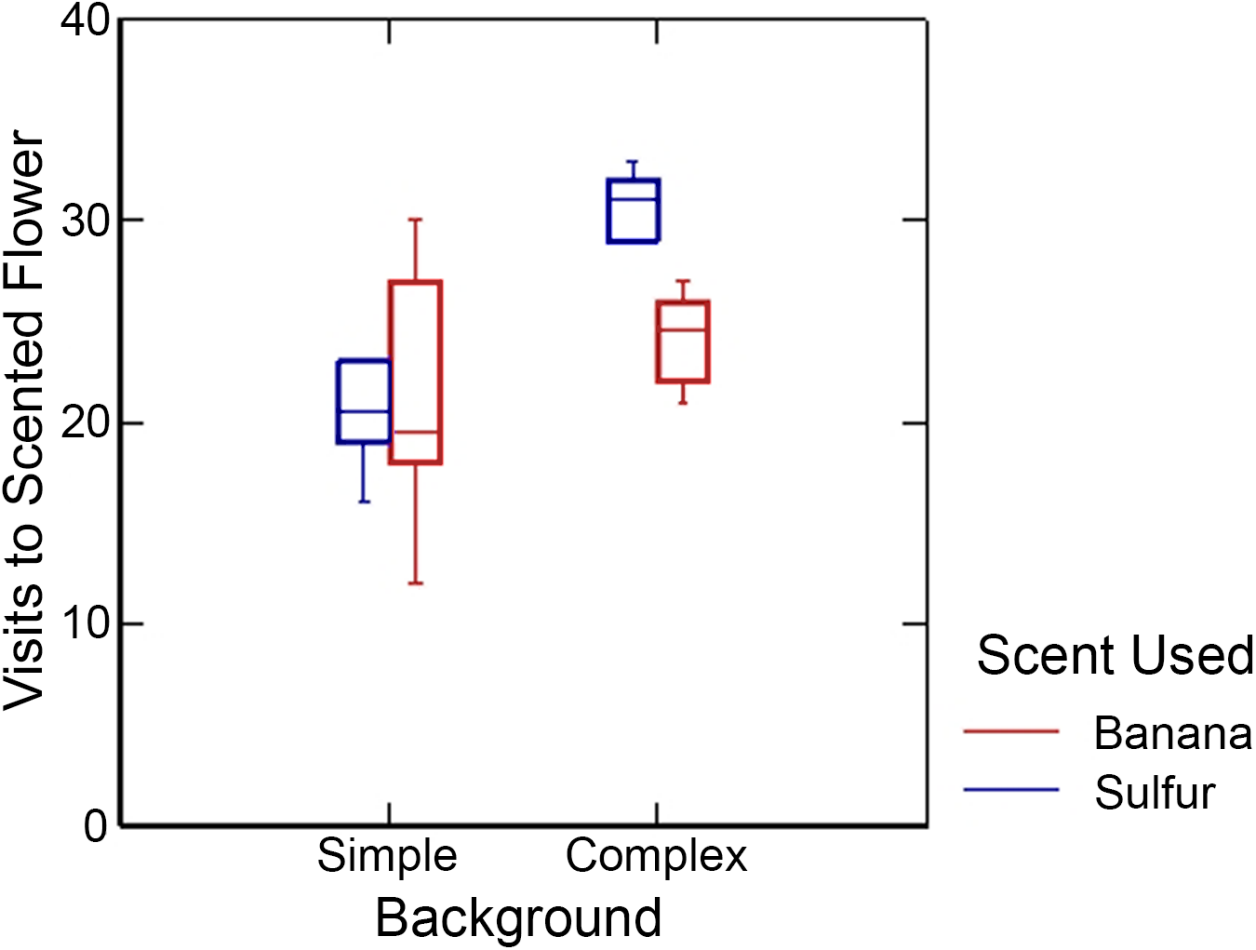
Interaction between Background and Scent. Number of visits, of the 40 total, to the flower with the training-flower scent (versus the one the training-flower shape) for sulfuric scent (blue) and banana scent (red) in simple and complex backgrounds.

Results of the GLM are summarized in Table 1. The bat species (*Anoura caudifer* or *A*. *geoffroyi*) and scent type (banana or dimethyl disulfide) used in the experiments did not significantly affect results. Background type showed a significant main effect: in simple backgrounds, bats visited the two flowers in similar proportions, while in complex backgrounds, bats visited the flower with the training-flower odor approximately twice as frequently as the flower with the training-flower shape (Fig 2). Trial number also showed a significant main effect: bats visited more scented flowers in the second set of twenty visits, after the 10-minute break, than in the first set of twenty visits (Fig 3). This increase in visits to scented flowers was particularly large in complex backgrounds and less apparent in simple backgrounds. Finally, there was a marginally significant interaction between background type and scent used: for the experiments with dimethyl disulfide there was a much steeper increase in reliance on scent in complex versus simple backgrounds than for the experiments with banana scent (Fig 4). None of the other two-way interactions were statistically significant, nor were the three-way or four-way interactions.

**Table 1:** General linear model results. Shows effects of various factors on the number of bat visits (out of 20 total visits) to the scented flower. These factors include bat species (*Anoura geoffroyi* or *A*. *caudifera*), scent type used (banana or sulfur), background (simple or complex), and trial number (first or second set of 20 visits). None of the 3-way and 4-way interactions were significant (results not shown).

Source		df	Mean Square	*F*	*P*	
**Between-subjects effects**						
	Intercept	1	6960.1	870.0	.000	
	Species	1	0.3	0.0	.843	
	Scent	1	27.0	3.4	.104	
	Species * Scent	1	0.1	0.0	.921	
	Error	8	8.0			
**Within-subjects effects**						
	Background	1	140.1	16.5	**.004**	**
	Background * Species	1	3.0	0.4	.569	
	Background * Scent	1	40.3	4.7	**.061**	†
	Error(Background)	8	8.5			
	Trial	1	21.3	10.2	**.013**	*
	Trial * Species	1	4.1	2.0	.199	
	Trial * Scent	1	0.1	0.0	.846	
	Error(Trial)	8	2.1			
	Background * Trial	1	5.3	2.3	.169	
	Error(Background*Trial)	8	2.3			

† *P* < 0.10

* *P* < 0.05

** *P* < 0.01.

## Discussion

Our results show that nectar bats shift to relying more on olfaction to locate flowers when background clutter makes floral shape a less reliable cue. In our experiments, bats first learned to locate a constantly-shifting flower with a specific shape and odor. When two flowers were subsequently presented to the bats, one with the previous odor and one with the previous shape, there were no statistical differences in which of the two flowers was located first when the flowers were presented in a simple background. However, when flowers were presented in a complex background with visual and acoustic ‘camouflage’, bats visited scented flowers approximately twice as frequently (Fig 2). Results were similar for *Anoura caudifer* and *Anoura geoffroyi*, and for banana or dimethyl disulphide scents; that is, there was no significant main effect for bat species or scent type (Table 1).

Why didn’t bats rely more heavily on olfaction in simple backgrounds? We argue that both flowers were readily apparent to bats under these conditions, and thus they essentially picked flowers at random. Anecdotally, we observed that bats in the simple-background experiments often visited whichever flower of the two was closer to its roosting position in the flight cage, and performed less looping, exploratory flights than bats in complex-background experiments. This observation supports the idea that locating the flowers did not pose as much of a challenge, and either vision or echolocation were sufficient. In complex backgrounds, the leaves and camouflage-colored sheet obscured the flowers, making shape a less reliable cue. The significant increase in visits to scented flowers suggests that the bats switched sensory modalities under these conditions and began to rely more on olfaction. The importance of background complexity has also been shown for bumblebees: Forrest and Thomson [33] found no preference for flower color in experiments with simple monochromatic backgrounds, and a significant preference for red in complex backgrounds with photographs of foliage.

Interestingly, the reliance on odor increased during the course of the experiments. Bats consistently visited less scented flowers in the first trial period of twenty repetitions than in the second trial period (after the 10 minute break; Fig 3). This increase was negligible for simple backgrounds and much larger in magnitude for complex backgrounds. This suggests that bats were learning to rely more heavily on olfaction during the complex-background experiments. This interpretation makes sense, given that the training phase had a simple background, and that the experiments in a simple background suggested shape was sufficient; bats only began to learn to rely more heavily on olfaction when faced with the more difficult task of locating the flower amidst clutter.

The actual odor used for the artificial flowers did not show a significant main effect. However, there was a marginally significant interaction between scent type and background type (P = 0.061; Table 1): there was a much steeper increase in visits to scented flowers in complex backgrounds when dimethyl disulphide rather than banana scent was used. Dimethyl disulphide is found in many bat-pollinated flowers [21, 23], including species that Ecuadorian *Anoura* are known to visit such as *Cleome anamola* and *Cobaea scandens* [34], and there is evidence that nectar bats possess an innate preference for this odor [23]. On the other hand, bat flowers are not known to produce banana-like scents, and nectar bats have likely never previously been exposed to this scent. Thus the scent X background interaction is likely because bats already associate sulphuric odors with food, both innately and through experience in nature. At the same time, the fact that bats still learn to seek out banana scent suggests that they can learn to associate any odor with food, even odors they have not previously been exposed to. A similar ability to quickly associate novel odors with food has also been shown for the frugivorous bat *Cynopterus sphinx* [35].

In nature, flowers always occur in some amount of background clutter. However, one trait common to chiropterophilous flowers is that they tend to be well-exposed beyond the plant’s foliage [20, 36], whether through flagelliflory (hanging inflorescences), cauliflory (inflorescences on trunks), or long pedicels (floral stems). The importance of floral exposure can be seen across the primarily bat-pollinated genus *Burmeistera*, for which the only species that does not possess long pedicels, *B*. *ceratocarpa*, instead has very small terminal leaves near its flowers [10]. Additionally, for *B*. *cyclostigmata* and *B*. *tenuiflora*, the greater the obstruction from undergrowth around individual flowers the less pollen the flowers received from bats at night [37]. Two adaptive explanations have been put forward to explain the importance of exposure for chiropterophilous flowers: 1) bats require open space around flowers because they sweep their wings in wide arcs in front of their bodies during hovering flight, while hummingbirds and insects do not 2) exposure separates flowers from background clutter and thus increases bat ability to detect flowers through vision or echolocation [20, 38]. Our results provide support for the latter hypothesis, in that bats readily located the well-exposed flowers in our simple backgrounds without needing to use olfaction. We also note that many bat-adapted flowers, such as *Cobaea trianae* and *Macrocarpea harlingii* [34], have evolved pedicels much longer than nectar bat wings, suggesting selection for long pedicels is not simply a response to hovering space requirements.

Our results parallel those of studies of frugivorous phyllostomids, which were found to rely on echolocation and olfaction in varying degrees depending on how well-exposed fruits are. *Phyllostomus hastatus* foraging on well-exposed, flagellichorous fruits of cucurbits, which dangle in open spaces away from foliage, rely primarily on echolocation to detect and localize fruits [39]. *Carollia* foraging on the somewhat well-exposed fruit spikes of *Piper* rely on odor for initial detection of fruiting branches, and then switch to echolocation for precise fruit localization [40]. At the opposite extreme, *Artibeus* and *Vampyressa* foraging for poorly-exposed figs nestled among leaves rely primarily on odor [41, 42]. Although fruit-eating bats clearly can use vision to aid in detecting food [43], the fact that bats still succeeding in finding food in total darkness in some of these experiments [40, 42] implies that olfactory and echoreflectance cues are sufficient.

In summary, this study found that the nectar bats *A*. *geoffroyi* and *A*. *caudifer* rely on both odor and shape to locate flowers when spatial cues are eliminated. Shape was apparently sufficient to locate flowers in backgrounds lacking clutter, but in complex backgrounds bats shifted to relying more on olfaction, and this use of olfaction increased over the course of the experiments. Flowers with dimethyl disulphide, a compound often found in bat flowers, were visited more frequently than those with banana scent. One interesting avenue for future research would be to study whether visual or acoustic cues were more important in locating flowers by shape, for example by recording vocalizations and by testing whether results change in differing light conditions, from dusk-like light levels to complete darkness. Additionally, our experiments were conducted at close distances (within the 2m^3^ flight cages); it would be interesting to explore whether reliance on different cues change when detecting flowers from greater distances. Finally, fruit-eating bats were found to learn novel food sources from odor cues in the breath and on the fur of roost-mates [44]; it is not known whether social information is similarly important for nectar-feeding bats. Do young bats learn appropriate flower odors from their mothers, or do they locate food sources by following adult bats? While the importance of spatial memory for foraging is clear (e.g. [13]), many questions remain regarding how nectar bats locate novel food sources in complex tropical habitats.

## Supporting Information

S1 Fig Artificial corolla shapes. Shapes and textures of the six artificial corollas used in the experiments, including cloth pentagons, plastic circles, foam squares (top row), fabric tubes, styrofoam triangles, and cardboard stars (bottom row).
(TIF)

S1 Table Study Sites. List of study sites with locations, elevations, fieldwork dates, and species used in the experiments (*Ageo = Anoura geoffroyi*, *Acau = A*. *caudifer*). All sites are located in Ecuador.
(DOCX)
