# Supplementary figures and images for: The Complexity of Background Clutter Affects Nectar Bat Use of Flower Odor and Shape Cues
Source: PLoS One. 2015 Oct 7;10(10):e0136657. doi: 10.1371/journal.pone.0136657 (PMC4596802; doi:10.1371/journal.pone.0136657)

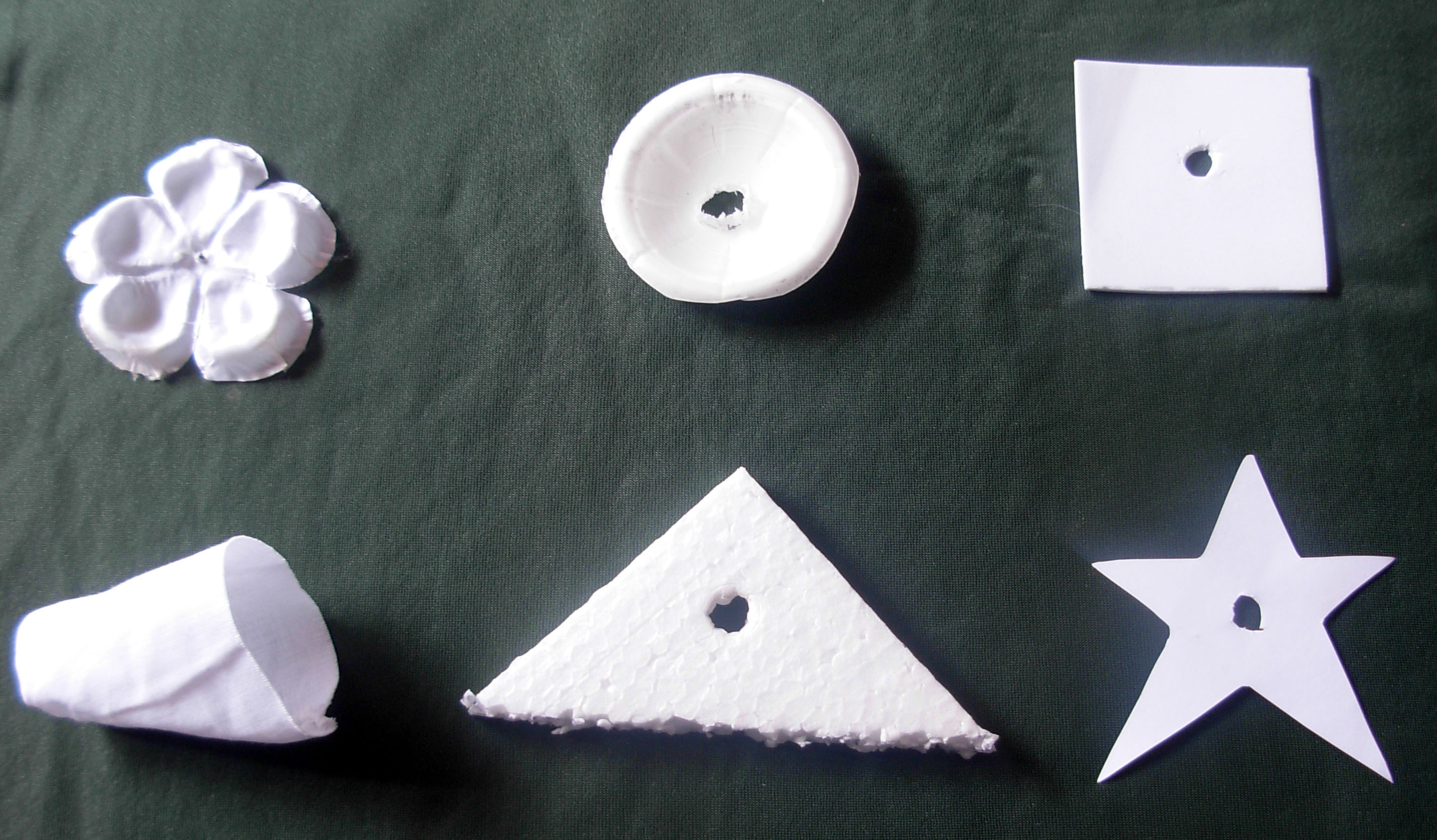

Supplement: S1 Fig — Shapes and textures of the six artificial corollas used in the experiments, including cloth pentagons, plastic circles, foam squares (top row), fabric tubes, styrofoam triangles, and cardboard stars (bottom row). (TIF) [file pone.0136657.s001.tif]
