# Supplementary material for: The Complexity of Background Clutter Affects Nectar Bat Use of Flower Odor and Shape Cues
Source: PLoS One. 2015 Oct 7;10(10):e0136657. doi: 10.1371/journal.pone.0136657 (PMC4596802; doi:10.1371/journal.pone.0136657)
Supplement: S1 Table — List of study sites with locations, elevations, fieldwork dates, and species used in the experiments (Ageo = Anoura geoffroyi, Acau = A. caudifer). All sites are located in Ecuador. (DOCX) [file pone.0136657.s002.docx]

**S1 Table.** **Study Sites.** List of study sites with locations, elevations, fieldwork dates, and species used in the experiments (*Ageo = Anoura geoffroyi*, *Acau = A. caudifer*). All sites are located in Ecuador.

| **Study Site (Province)** | **Location** | **Elevation (m.a.s.l.)** | **Fieldwork Dates** | **Experiments** |
| --- | --- | --- | --- | --- |
| Yanayacu (Napo) | 0º35'S,77º52'W | 2000-2500 | Jul. 16- 28, 2010 | 2 *Acau* |
| Domono (Morona-Santiago) | 2º13'S,78º07'W | 1100-1800 | Oct. 2-14, 2010 | 1 *Ageo* |
| Tapichalaca (Loja) | 4º29'S,79º07'W | 1800-3100 | Nov. 4-16, 2010 | 1 *Acau*, 2 *Ageo* |
| Guajalito (Pichincha) | 0º13'S,78º48'W | 1800-2300 | Jan. 24-31, 2011 | 1 *Acau*, 3 *Ageo* |
| Hacienda Carolina (Napo) | 0º25'S,77º51'W | 1600-1900 | Jan. 7-14, 2011 | 2 *Acau* |
